# A Fiber-Based Chromatic Dispersion Probe for Simultaneous Measurement of Dual-Axis Absolute and Relative Displacement

**DOI:** 10.3390/s22249906

**Published:** 2022-12-16

**Authors:** Ran Zhao, Chong Chen, Xin Xiong, Yuan-Liu Chen, Bing-Feng Ju

**Affiliations:** 1 The State Key Laboratory of Fluid Power and Mechatronic Systems, Zhejiang University, Hangzhou 310058, China; 2 Hangzhou Global Scientific and Technological Innovation Center, Zhejiang University, Hangzhou 310027, China

**Keywords:** fiber-based probe, chromatic dispersion, dual-axis displacement measurement, absolute and relative displacement measurement, nanometric resolution

## Abstract

This paper presents a fiber-based chromatic dispersion probe for the simultaneous measurement of dual-axis absolute and relative displacement with nanometric resolutions. The proposed chromatic dispersion probe is based on optical dispersion. In the probe, the employed light beam is split into two sub-beams, and then the two sub-beams are made to pass through two optical paths with different optical settings where two identical single-mode fiber detectors are located at different defocused positions of the respective dispersive lenses. In this way, two spectral signals can be obtained to indicate the absolute displacement of each of the dual-axes. A signal processing algorithm is proposed to generate a normalized output wavelength that indicates the relative displacement of the dual-axis. With the proposed chromatic dispersion probe, the absolute and relative displacement measurements of the dual-axis can be realized simultaneously. Theoretical and experimental investigations reveal that the developed chromatic dispersion probe realizes an absolute measurement range and a measurement resolution of approximately 180 μm and 50 nm, respectively, for each axis. Moreover, a relative displacement measurement range and a measurement resolution of about 240 μm and 100 nm, respectively, are achieved for the dual-axis.

## 1. Introduction

Precision displacement measurement is a crucial goal in various fields of nanotechnology systems, including precision optics [1,2,3,4], semiconductor manufacturing [5,6], optical direct writing systems, and CNC machine tools [7,8,9]. Meanwhile, the demand for multi-axes positioning is also increasing. To meet the need for precise displacement measurements with sub-micrometric accuracy, many traditional displacement measurement techniques [10,11,12,13,14] are available such as laser interferometers [10,11], optical encoders [12], and capacitive displacement sensors, as well as confocal probes [13,14].

However, when the size of the measured object drops to the micron level, challenges are generated for the above-mentioned displacement measurement technologies, as the displacement calibration devices are under space-limited and small-cavity conditions [15,16]. To address this issue, various displacement measurement techniques have been proposed [17,18,19,20]. A miniature laser diode interferometer has been proposed for investigating the effects of the retroreflector’s motion error on measurement accuracy [17]. Fabry-Pérot interferometers have been established and verified as another type of interferometer for displacement measurement in space-limited conditions [18,19,20]. However, the interferometer is sensitive and fragile to environmental disturbances such as temperature, humidity, pressure, air turbulence, and so on, which change the air refractive index within the long optical path of the interferometer, and it is also complicated to mount [21,22,23]. Moreover, reference mirror error is difficult to eliminate [24]. To deal with the defects of the laser interferometer, Cr-N thin film displacement sensors have been developed and can be installed on a micro-stage, but due to the existence of electronic components within the sensors, electrical and electromagnetic interference are the main drawbacks of these sensors [12,25,26,27,28].

Meanwhile, with the development of fiber optics, optical fiber has been widely used in displacement measurement sensors with its remarkable characteristics of anti-electromagnetic interference, corrosion resistance, portability, easy installation, and geometric flexibility [29,30,31,32,33,34]. An optical fiber sensor based on the macro-bending effect has been proposed for dual-axis displacement measurement [30,31]. A differential optical-fiber displacement sensor has been proposed for one-axis displacement measurement [32]. A fiber-based interferometer has also been proposed for single-axis displacement measurement [33,34]. In the above fiber-based displacement measurement techniques, more than one detector is required to obtain the dual-axis displacement. More importantly, the absolute displacement of the single-axis and relative displacement between the dual-axis cannot be obtained simultaneously. This is one of the key problems that impede the practical application of the traditional fiber-based displacement measurement techniques in single- and dual-axis displacement measurements under space-limited and small-cavity conditions.

Therefore, to address the above-mentioned issues, in this paper, a fiber-based chromatic dispersion probe for the simultaneous measurement of the dual-axis absolute and relative displacement has been developed. The proposed chromatic dispersion probe is based on optical dispersion. In the probe, the employed light beam is split into two sub-beams that are then made to pass through two optical paths with different optical settings. Two identical single-mode fiber detectors are placed at different defocused positions of the dispersive lenses to obtain two spectral signals. The two spectral signals are used to evaluate the absolute displacement of the dual-axis. A signal processing algorithm is proposed to generate a normalized output wavelength that indicates the relative displacement of the dual-axis. With the proposed chromatic dispersion probe, the absolute and relative displacement measurements of the dual-axis can be realized simultaneously.

The remainder of this paper is organized as follows: Firstly, the optical transfer function of the system is calculated based on the Fresnel diffraction principle, and the measurement principle together with the signal processing algorithm and data fitting method for the absolute and relative displacement measurement of the proposed dual-axis chromatic dispersion probe is introduced, as presented in Section 2. Secondly, a system description and simulation of the absolute and relative displacement measurement of the established chromatic dispersion probe are presented in Section 3. Finally, experiments are carried out to verify the validity and feasibility of the established chromatic dispersion probe, and the experiment results are presented in Section 4.

## 2. Principle

### 2.1. Chromatic Dispersion and Principle

**Figure 1:**
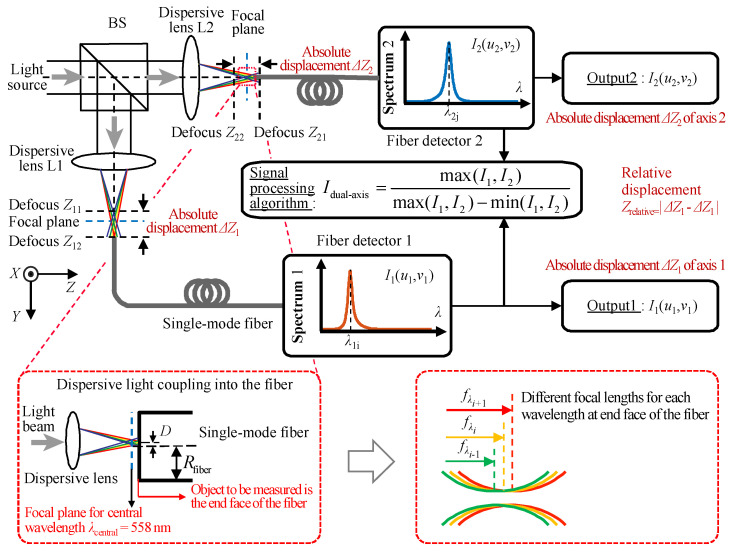
Schematic of the optical configuration for the established fiber-based chromatic dispersion probe.

**Figure 2:**
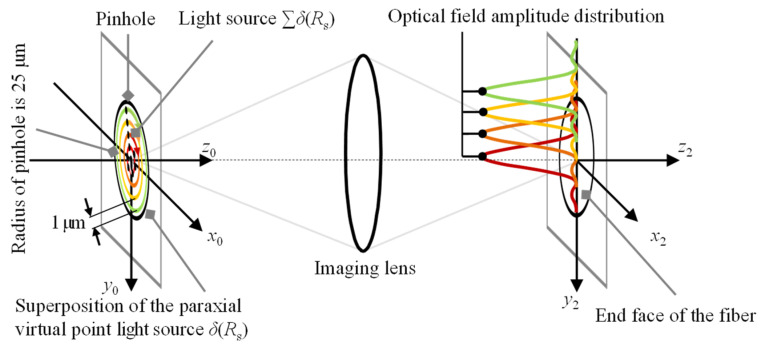
Schematic diagram of the superposition principle of the paraxial point light sources.

Figure 1 depicts a schematic diagram of the configuration of the established fiber-based chromatic dispersion probe for the simultaneous measurement of dual-axis absolute and relative displacement. It should be noted that in Figure 1, the object to be measured is the end of two single-mode optical fibers. It should also be pointed out that the focal plane in Figure 1 is one of many continuous focal planes of the chromatic dispersion system and corresponds to the central wavelength of the dispersive lenses. The fiber-based chromatic dispersion probe proposed in this paper is fundamentally different from the confocal configurations. Due to the remarkable geometric flexibility and micro size of the optical fiber, it can be easily integrated into the linear stage to be measured, and the displacement of the stage for measurement can be carried out by measuring the displacement of the end face of the optical fiber. In the fiber-based chromatic dispersion probe, two sub-beams are focused in two different directions through a beam splitter and can be used to measure the absolute displacement of two micro stages in two optical paths with prior calibration. In this paper, dual-axis relative displacement refers to the relative displacement of two independent objects in two different directions. The proposed dispersion probe can measure the absolute displacement of two linear stages and the relative displacement between the two independent stages simultaneously, which is crucial for position feedback and the calibration of the microscale stages under space-limited and small-cavity conditions such as the relative displacement of two reflective mirrors in the optical resonant cavity and the Michelson interferometer, where traditionally only one reflective mirror can be moved and its absolute displacement can be calibrated, which will greatly limit the usage scenarios and application scope. Single-mode optical fibers are employed as the signal-transferring media between the optical spectrometer and the fiber detectors in which the employment of the optical fibers in the chromatic dispersion probe, instead of a traditionally utilized pinhole, can ensure a convenient and coherent optical system for signal detection and acquisition regardless of the detailed optical light paths and the fiber dimensions [35,36]. In an ideal optical system, the light beam passing through a small pinhole can be regarded as a point light source and can further be simplified to the impulse function of *δ*(0), but in practical applications, to ensure a better signal-to-noise ratio for the spectrum, the size of the pinhole needs to be appropriately increased, making it difficult to simplify it into a simple point light source [36,37]. In our dispersion probe system, the light beam passing through a small pinhole is regarded as the superposition of the paraxial point light sources, which means the superposition of the impulse functions of ∑*δ*(*R*_s_). Figure 2 depicts the principle of the superposition of the paraxial point light sources in which the radius of the light source is 25 μm, corresponding to the radius of the pinhole, and by multiplying the area of the ring (with a width of 0.5 μm) by the pulse function, the light source model is finally obtained by superposing a ring of pulse functions every 1 μm from the center of the light source. The point spread functions of all virtual point sources on the detection surface can be calculated, respectively, which are summed up to obtain the amplitude distribution and acquire the spectral distribution. It should be noted that two single-mode fibers are employed as the probe, which is equivalent to the point detectors, and so the imaging process is considered to be coherent. Therefore, the amplitude distribution of the total optical field detected by the fiber probe is the superposition of the amplitudes contributed by the samples at different points [38], which yields the received light intensity distribution given by Equation (1). The light distribution of the established dispersion probe system can be given as

(1) $$I(u,v)=|\sum_{R_{s}}h_{\mathrm{eff}}(u,v){|}^{2}.$$

in which *R*_s_ is the distance between the light source center and the superimposed paraxial point light sources, *h*_eff_ denotes the effective point spread function of the fiber-based optical system, and *u* and *v* are optical coordinates related to the actual defocus distance *Z* and off-axis distance *D*, which are given by [36,37]

(2) $$u=\frac{2\pi }{\lambda }Z\sin^{2}\alpha ,$$

(3) $$v=\frac{2\pi }{\lambda }(D-\frac{R_{s}}{M})\sin\alpha .$$

where sin *α* is the numerical aperture (NA) of the employed chromatic dispersion lens and *M* is the magnification of this optical system.

The effective point spread function is obtained by convolving the point spread function of the optical system and the fundamental mode of the single-mode fiber, which is given by

(4) $$h_{\mathrm{eff}}=h_{m}(u,v)⊗e(v).$$

where *h*_m_(*u*, *v*) represents the point spread functions for the optical system, whose sub-scripts indicate two different measurement axes, and *e*(*v*) stands for the eigenfunction of the fundamental mode of the single-mode fiber [39].

**Figure 3:**
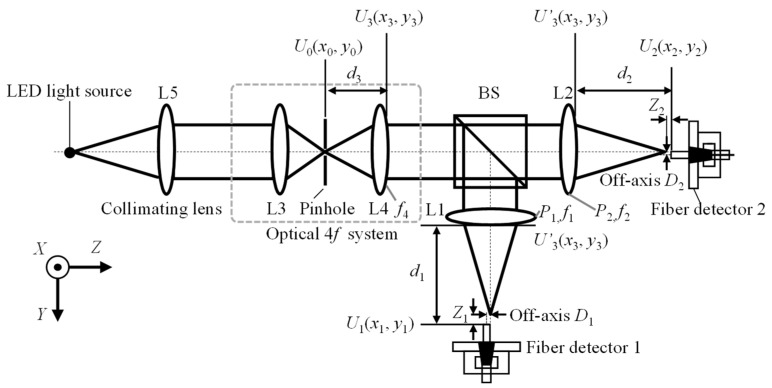
Schematic of the optical measurement system of the proposed fiber-based chromatic dispersion probe.

Firstly, the point spread function of the optical system is calculated. Figure 3 shows the optical structure of the proposed dispersion probe and analyzes the imaging process. Four Cartesian coordinate systems are established in the pinhole plane. The end face of the two fibers and the plane just behind lens L4 are denoted as *x*_j_, *y*_j_, and *z*_j_ (j = 0, 1, 2, and 3), respectively, and are shown in Figure 3. According to the Fresnel diffraction principle of the lenses [37,38], the point spread function of this optical system is shown in Equation (5):

(5) $$U_{m}(x_{m},y_{m})=A\iint P_{m}(x_{m},y_{m})e^{\frac{jk}{2}(\frac{1}{f_{4}}+\frac{1}{f_{m}}-\frac{1}{d_{3}}-\frac{1}{d_{m}})}e^{jk[(\frac{x_{0}}{d_{3}}+\frac{x_{m}}{d_{m}})x_{3}+(\frac{y_{0}}{d_{3}}+\frac{y_{m}}{d_{m}})y_{3}]}dx_{3}dy_{3},m=1,2,$$

where *f*_m_ is the focal length corresponding to the central wavelength of the dispersive lenses L1 and L2, whose sub-scripts indicate two different measurement axes, respectively, *f*_4_ is the focal length of the achromatic lens L4, *d*_3_ is the distance between the pinhole and the achromatic lens L4, and *d*_m_ is the distance between the end face of the fiber and the dispersive lens L1 and L2. It should be noted that *d*_m_ = *f*_m_ + *Z*_m_. *Z*_m_ is the defocus distances of the two different measurement axes. *P*_m_(*x*_m_*, y*_m_) is the pupil function of the corresponding dispersive lenses L1 and L2, which can be expressed by

(6) $$P_{m}(x_{m},y_{m})=\{\begin{array}{cc} 1 & \sqrt{x_{m}{}^{2}+y_{m}{}^{2}}\leq a\\ 0 & \sqrt{x_{m}{}^{2}+y_{m}{}^{2}}>a \end{array},m=1,2,$$

where *a* is the radius of the dispersive lenses L1 and L2.

The amplitude distribution for the end face of the single-mode optical fiber is shown in Equation (7), which brings Equations (2), (3), and (6) into Equation (5):

(7) $$U_{m}(x_{m},y_{m})=A’\int_{0}^{1}P_{m}(r)e^{-\frac{ju}{2}r^{2}}J_{0}(vr)dr,m=1,2,$$

where *J*_0_ is a zero-order Bessel function of the first kind and *A*′ is the coefficient before the integral term that can be omitted in this paper.

Therefore, the point spread function of the optical system is shown in Equation (8), in which *P*_m_(*u*, *v*) are the pupil functions of the corresponding dispersive lenses:

(8) $$h_{m}(u,v)=\int_{0}^{1}P_{m}(u,r)J_{0}(vr)rdr,m=1,2.$$

Secondly, the fundamental mode of the single-mode fiber is taken into consideration. For a single-mode fiber, the electric field vector at the incidence of the fiber can be expressed as an eigenfunction expansion by [36,40]

(9) $$e=\sum {\vec{e}}_{n},$$

(10) $$\vec{e_{n}}=\hat{x}\frac{f_{n}(R)}{\sqrt{\psi_{n}}}\cos(n-1)\varphi ,$$

where *ψ_n_* is the direction angle of the electric vector, *φ* represents the polarization angle, and *n* stands for the *n*th mode of the employed fiber and equals 1 in this paper. *f_n_*(*R*) stands for the solution of the transverse electromagnetic field and can be expressed as [40,41]

(11) $$f_{n}(R)=\{\begin{array}{c} \frac{J_{n-1}(UR)}{J_{n-1}(U)},R\leq 1\\ \frac{K_{n-1}(WR)}{K_{n-1}(W)},R>1 \end{array}.$$

where *U* and *W* are the scalar modal parameters for the core and cladding of the fiber, respectively, which can be found in [39] (page 314).

The axial responses of the two measurement axes can be expressed by bringing Equations (4) and (8)–(11) into Equation (1) and are given by Equation (12):

(12) $$I_{m}(u_{m},v_{m})={\left|\sum_{R_{s}}A\int_{0}^{1}e^{-\frac{ju_{m}}{2}r^{2}}J_{0}(v_{m}r)\frac{V^{2}\left[rv_{c}J_{1}(rv_{c})-J_{0}(rv_{c})G(U)\right]}{\left({\left(rv_{c}\right)}^{2}-U^{2}\right)\left({\left(rv_{c}\right)}^{2}+W^{2}\right)}rdr\right|}^{2},m=1,\;2.$$

where *R*_fiber_ is the radius of the core of the single-mode optical fiber, *R* is the ratio of the off-axis distance *D* to the radius of the fiber, *R* = *D*/*R*_fiber_, and *v*_c_ represents the optical coordinate and is given by 2π *R*_fiber_ sin *α*/*λ*, *J*_1_(*rv*_c_), which is the first order Bessel function of the first kind. The function *G*(*U*) is expressed as

(13) $$G(U)=\frac{UJ_{1}(U)}{J_{0}(U)}.$$

According to the thick-lens equation, the focal length formula is given by [36,37]

(14) $$f_{\lambda }=\frac{1}{\left(n_{\lambda }-1\right)\left(1/R_{1}-1/R_{2}\right)+\left(n_{\lambda }-1\right)d/\left(n_{\lambda }R_{1}R_{2}\right)},$$

where *R*_1_ and *R*_2_ stand for the curvature radii of the lens, *d* is the thickness of the lens, and *n_λ_* is the refractive index of the lens material and can be characterized by the Sellmeier equation [42]:

(15) $$n_{\lambda }=\sqrt{1+\frac{B_{1}\lambda^{2}}{\lambda^{2}-C_{1}}+\frac{B_{2}\lambda^{2}}{\lambda^{2}-C_{2}}+\frac{B_{3}\lambda^{2}}{\lambda^{2}-C_{3}}},$$

where *B*_i_ and *C*_i_ (i = 1, 2, 3) are the Sellmeier coefficients.

**Figure 4:**
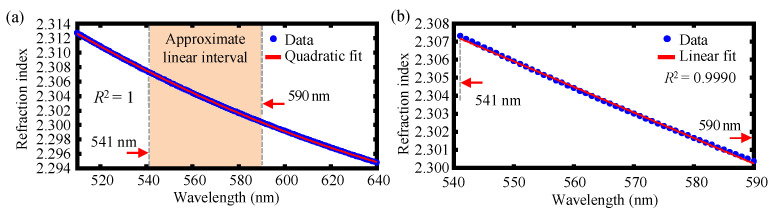
The relationship between refractive index *n_λ_* and wavelength *λ* (**a**) by quadratic fit and (**b**) linear fit in the working spectrum range for the dual-axis absolute and relative displacement measurement.

Figure 4 represents the relationship between the refractive index *n_λ_* and the wavelength *λ* by the quadratic fit and the linear fit in the working spectrum range for the dual-axis absolute and relative displacement measurement according to Equation (15). It can be observed that *n_λ_* shows a quadratic polynomial in the available spectral range depicted in Figure 4a and can be given by

(16) $$n_{\lambda }=k_{1}\lambda^{2}+k_{2}\lambda +b.$$

where *k*_1_, *k*_2_, and *b* are the quadratic coefficients, slope, and intercept, respectively. The quadratic polynomial fitting result is *k*_1_ = 3.3725 × 10^−7^ nm^−2^, *k*_2_ = −5.2468 × 10^−4^ nm^−1^, and *b* = 2.4925, with the coefficient of determination *R*^2^ being approximately 1. The quadratic coefficient is much smaller than the linear coefficient, so *n*_λ_ has a good linear relationship with the wavelength *λ*, which is locally demonstrated in Figure 4b. The absolute displacement for each axis is given by

(17) $$\Delta Z_{m}=f_{\lambda }-f_{\lambda_{0}}(m=1,2),$$

where *f_λ_*_0_ is the focal length of the wavelength of an arbitrary initial position and *f_λ_* is the focal length of the wavelength *λ*, which is the peak wavelength of the spectrum that corresponds to a specific distance *Z* from the focal plane. Δ*Z*_m_ (m = 1, 2) represents the absolute displacement of the two axes; therefore, the relative displacement *Z*_relative_ can be given by

(18) $$Z_{\mathrm{relative}}=\left|\Delta Z_{1}-\Delta Z_{2}\right|,$$

According to Equations (14) and (17), the following differential equation can be obtained and is given by Equation (19):

(19) $$dZ=df_{\lambda }=-\frac{dn_{\lambda }}{n_{\lambda }-1}\cdot f_{\lambda },$$

By substituting Equations (14) and (16) into Equation (19), the sensitivity of the absolute displacement measurement can be approached and is given by Equation (20):

(20) $$\frac{d\lambda }{dZ}=-\frac{k_{1}\lambda^{2}+k_{2}\lambda +b-1}{2k_{1}\lambda +k_{2}}\cdot \frac{1}{f_{\lambda }}=-(\frac{\lambda }{2}+\frac{k_{2}}{4k_{1}}+\frac{b-1-k_{2}{}^{2}/4k_{1}}{2k_{1}\lambda +k_{2}})\frac{1}{f_{\lambda }}\approx -\frac{b-1-k_{2}{}^{2}/4k_{1}}{2k_{1}\lambda_{0}+k_{2}}\frac{1}{f_{\lambda_{0}}}.$$

It should be pointed out that even though the relationship between *n_λ_* and *λ* has a tiny quadratic term, the estimation of the measurement sensitivity at any given central wavelength *λ*_0_ can be carried out. As is shown in Figure 4b, the working spectral range of 541 nm to 590 nm yields good linearity, so we can estimate the measurement range by using the linear relationship between the *n_λ_* and *λ*. The measurement sensitivity varies slightly at different wavelengths. Since the light beam passes through the pinhole and is regarded as the superposition of different paraxial point light sources, the difference between the sensitivity results of the employed light source and the calculation results of an ideal point light source can be observed. It should also be noted that the theoretical calculation method in this paper takes into consideration the tiny quadratic term in the dispersion relationship, which can estimate the sensitivity over a larger range compared with a method that considers the linear term only. Moreover, this theoretical calculation method can also reduce the design difficulty of the whole system and expand the measurement range of the probe in this paper.

### 2.2. Signal Processing Algorithm and Data Fitting Method

To obtain the relative displacement of the dual-axis simultaneously, a signal processing algorithm is proposed to generate a normalized output wavelength that indicates the relative displacement of the dual-axis. The signal processing algorithm *I*_dual-axis_ is expressed as

(21) $$I_{\mathrm{dual}-\mathrm{axis}}=\frac{\max(I_{1},I_{2})}{\max(I_{1},I_{2})-\min(I_{1},I_{2})}.$$

It should be pointed out that the signal processing algorithm proposed in this paper is inspired by a signal processing method used in our previous work [43] but with a different calculation formula. More importantly, we should note the differences in the theory and significance when compared with [43]. The signals detected by the two fiber detectors are dispersive spectral signals. From the point of view of the function of the signal processing algorithm proposed in this paper, the relative displacement of the dual-axis can be obtained. Meanwhile, the signals in [43] are confocal signals with different positions of the reference mirror, and the signal processing method in [43] is used to determine the displacement of the reference mirror, which is the measured object, not the absolute or relative positions of the two fiber detectors. Moreover, according to Equations (1) and (12), *I*_1_ and *I*_2_ are newly derived equations standing for the dispersion spectral signals generated from a light source superposed by virtual point light sources, while *I*_1_ and *I*_2_ in [43] are only confocal spectral signals. In addition, for the relative displacement measurements of the two single-mode fibers positioned in two axes, the fitting and height matching methods are also needed in the proposed signal processing algorithm in our manuscript. From the perspective of the principle of the formula of the proposed signal processing algorithm, the denominator of the formula no longer requires an absolute value operation in [43], and the numerator of the formula is no longer the sum of the two signals, which simplifies the signal processing and saves signal processing time. It is also mentioned in our manuscript that the proposed signal processing algorithm for calculating the relative displacement of the two axes can be mainly divided into three steps. The first step is the 8-order sum of Sin fitting, the second step is the two-axis 98% peak matching, and the third step is to use Equation (21) to obtain *I*_dual-axis_ and to use the peak wavelength of the normalized *I*_dual-axis_ to obtain the relative displacement of the two single-mode fibers positioned in two axes.

**Figure 5:**
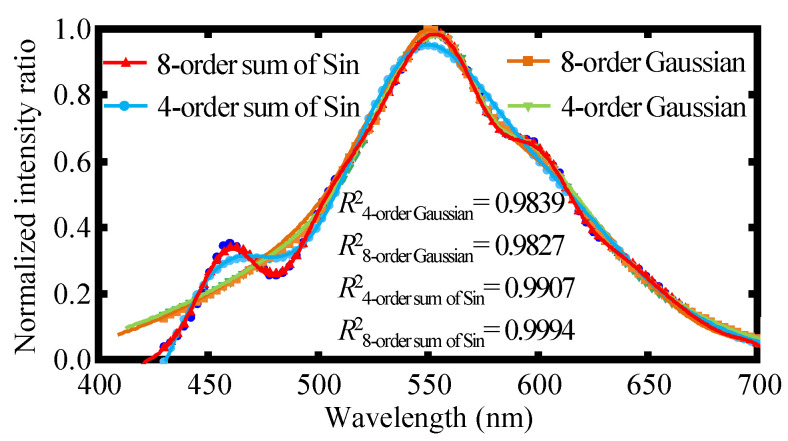
Comparison of the effects of various fitting methods including the 8-order and 4-order sum of Sin and the 8-order and 4-order Gaussian on the obtained experimental spectrum.

**Figure 6:**
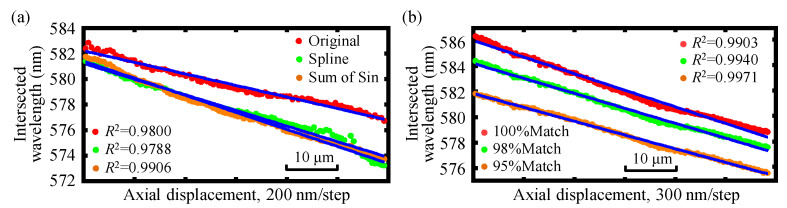
Comparison of different (**a**) data fitting methods and (**b**) the corresponding peak matching for the dual-axis absolute displacement measurement.

In actual experiments, due to the influence of light source stability, temperature, and humidity, there is spectral instability. When measuring the relative position of the two axes, there is a problem with multi-intersection aliasing, which affects the measurement range and measurement resolution. To address the above defects, different data fitting methods are employed in the signal data processing, and it is found that the Spline and the sum of Sin used in the signal data fitting can effectively solve the problem of multi-intersection mixing and lead to the measurement range expansion together with the resolution enhancement. As is shown in Figure 5, due to the irregular shape of the spectrum, the most commonly used Gaussian function is not satisfactory. It is obvious that the 8-order sum of the Sin function can obtain the best-fitting results. It should be emphasized that there are no discontinuities in the spectrum, so no Gibbs oscillation exists in the fitting procedure, as depicted in Figure 5. The signal data fitting results are shown in Figure 6a, from which the employed 8-order sum of Sin method can achieve better linearity and measurement range. Moreover, since the spectrum of the employed LED light source is non-smooth and the spectral signal peaks of the two axes obtained from the two fiber detectors cannot be matched well, it is necessary to perform peak matching during data processing at the intersections of spectral positions with better linearity. The results are presented in Figure 6b, from which it can be observed that 98% peak matching is sufficient to improve the measurement range and resolution with good linearity for the proposed chromatic dispersion probe. It should be noted that “200 nm/step” in Figure 6 means that the movement step of the fiber detector is 200 nm in the experiment.

### 2.3. Measurement Principle

#### 2.3.1. Measurement Principle of the Absolute Displacement of Each of the Dual-Axes

**Figure 7:**
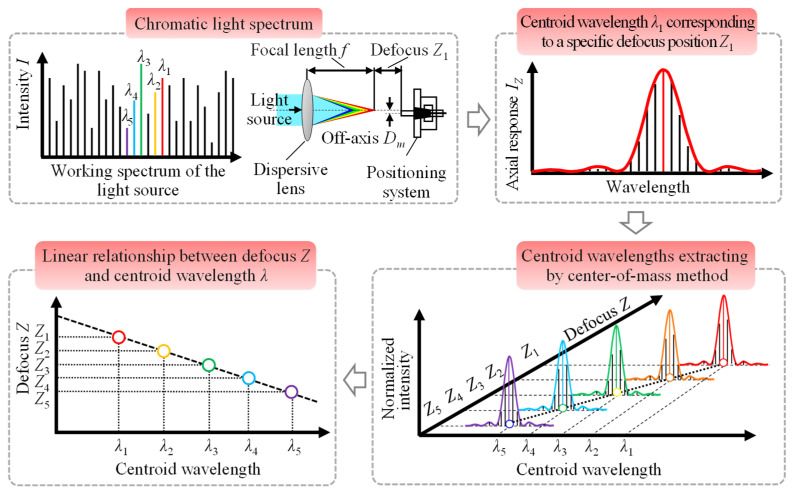
Flow chart of the measurement principle and decoding process for the absolute displacement measurement of each of the dual-axes of the proposed fiber-based chromatic dispersion probe.

Figure 7 depicts a flow chart of the measurement principle and decoding process for the absolute displacement measurement of each of the dual-axes of the proposed chromatic dispersion probe. It should be pointed out that in actual experiments, due to the influence of the light source stability, temperature, and humidity, there is spectral instability, and thus the centroid wavelength method is employed in the decoding process of both the simulation and the experiment. After the light source with a chromatic light spectrum passes through the designed optical path, the fiber detector at a specific position *Z* obtains a corresponding axial response spectrum *I*_z_. It should be noted that the normalization method of the spectrum is that each wavelength in the axial response spectrum is divided by the maximum light intensity that is chosen as the normalized criterion. The wavelength *λ* is the centroid wavelength of the obtained spectral signal, which is extracted by the center-of-mass method from the axial response spectrum. At different defocus distances, *Z*_1_, *Z*_2_, *Z*_3_, …, different axial response spectra of *I*_1_, *I*_2_, *I*_3_, … that correspond to different centroid wavelengths of *λ*_1_, *λ*_2_, *λ*_3_, …. can be approached. The absolute displacement can be acquired by decoding the linear relationship between the defocusing distance *Z* and the centroid wavelength *λ*. By using a mechanical optical switch, the spectral information of the dual-axes can be detected simultaneously. As a result, the measurement of the absolute displacement information of the dual-axes can be realized synchronously.

#### 2.3.2. Measurement Principle of the Dual-Axis Relative Displacement

**Figure 8:**
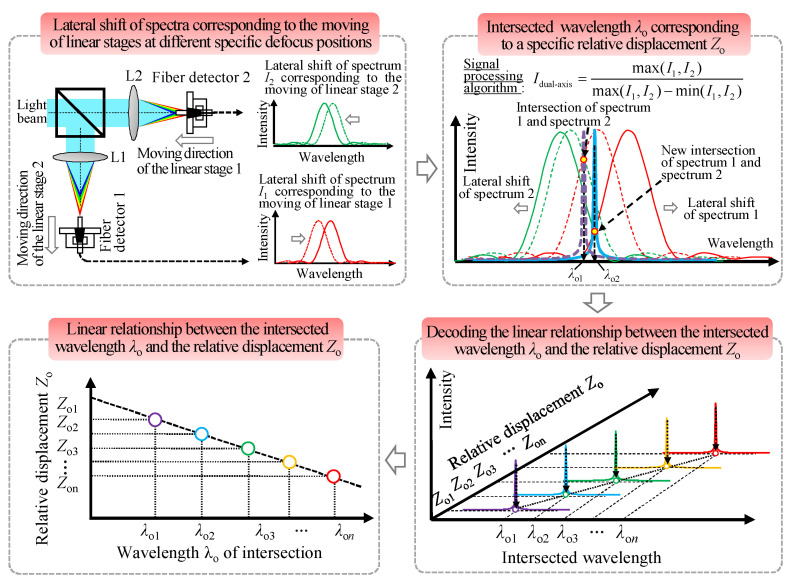
Flow chart of the measurement principle and decoding process for the relative displacement measurement of the dual-axis of the proposed fiber-based chromatic dispersion probe.

Figure 8 shows a flow chart of the measurement principle and decoding process for the relative displacement measurement of the dual-axis of the proposed chromatic dispersion probe. To obtain the relative displacement of the dual-axis of the established chromatic dispersion probe, a signal processing algorithm *I*_dual-axis_ is proposed in which spectrum 1 corresponds to a specific defocusing distance in fiber detector 1 and spectrum 2 is relevant to another specific defocusing distance in fiber detector 2. The signal processing algorithm consists of two steps: first, the *I*_dual-axis_ is calculated according to Equation (21), and then it is normalized using its maximum light intensity. Hence, the intersected wavelength *λ*_o1_ is related to the intersection of spectrum 1 (dotted green line), and spectrum 2 (dotted red line) can be obtained. Furthermore, with the moving of the fiber detectors by the two linear stages, the lateral shift of spectrum 1 and 2 corresponding to the moving of linear stages 1 and 2, respectively, can be observed. The signal processing algorithm is used to obtain *I*_dual-axis_ (solid blue line) corresponding to the new intersection of the laterally moved spectrum 1 (solid green line) and the laterally moved spectrum 2 (solid red line). The intersected wavelength *λ*_o2_ is relevant to the relative displacement of the dual-axis. Therefore, by decoding the linear correspondence between the spectral intersected wavelength *λ*_o_ and the relative displacement *Z*_o_ of the dual-axis, the *Z*_o_ of the dual-axis of the established chromatic dispersion probe can be extracted, as illustrated in Figure 8.

## 3. System Description and Simulation

### 3.1. Instrumental Configuration

**Figure 9:**
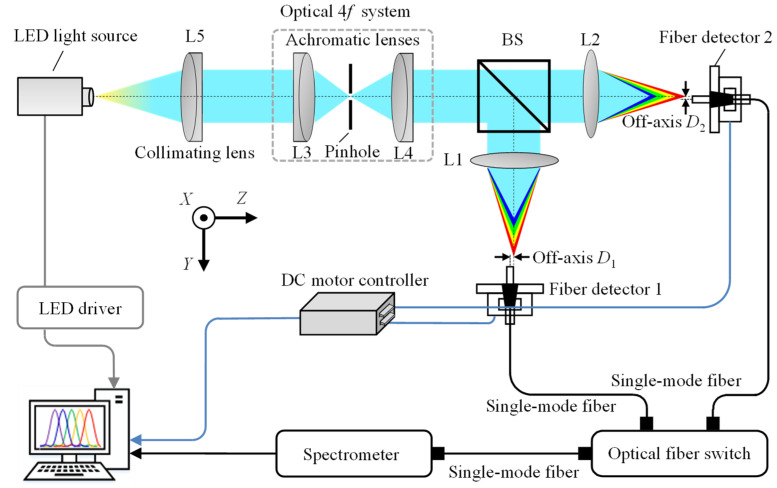
Schematic diagram of the prototype of the established optical configuration of the fiber-based chromatic dispersion probe for the simultaneous measurement of the dual-axis absolute and relative displacement.

**Figure 10:**
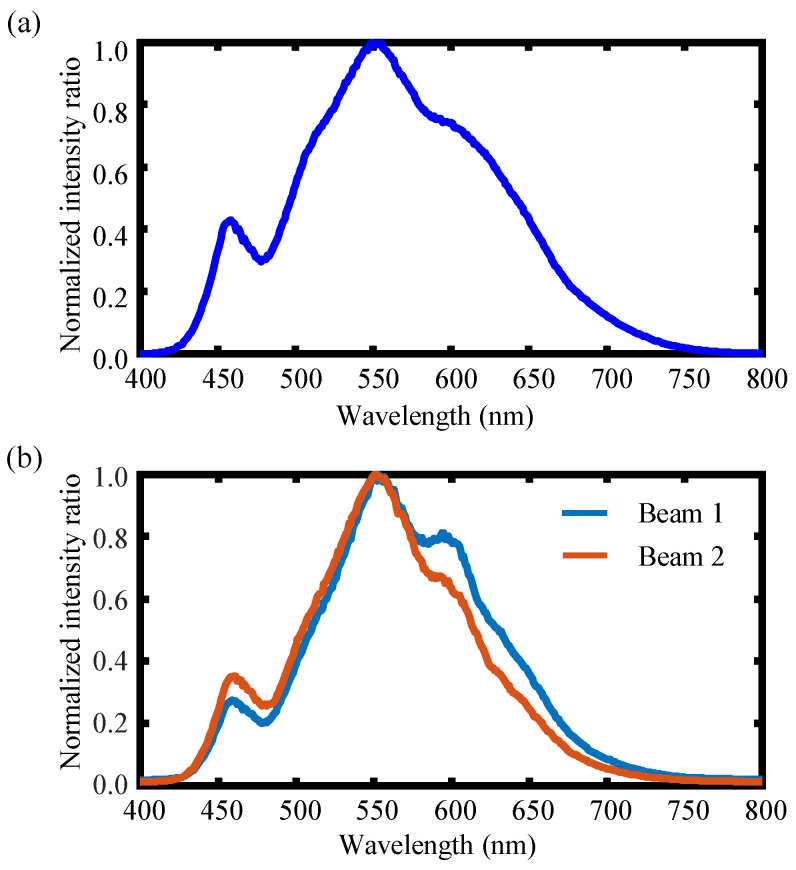
The spectrum of (**a**) the employed LED light source and (**b**) two detected spectral signals of the dual-axis obtained by the two fiber detectors at the focal planes of the two dispersion lenses L1 and L2.

Figure 9 describes the schematic optical configuration of the established chromatic dispersion probe for the dual-axis absolute and relative displacement measurement. An LED illuminator (Lumileds, San Jose, United States, L1CU-4090 ) with a homemade power supply circuit was employed as the chromatic light source. A beam was emitted from the chromatic light source with a working spectrum ranging from 400 nm to 800 nm, and it was collimated by a collimator lens L5 (Thorlabs Inc., Newton, New Jersey, United States, AC254-030-A-ML) to generate a collimated beam that was then made to pass through an optical 4*f* system in which a pinhole with a diameter of 50 µm was employed and located on the focal planes of the two achromatic lenses L3 and L4. The beam passing through the pinhole could be filtered, and the high-frequency noise could be eliminated effectively. After passing through the 4*f* system, the light beam was then divided into two sub-beams by an unpolarized beam splitter (BS). After passing through the two dispersive lenses L1 and L2, the two sub-beams were received by two identical fiber detectors, which were composed of two single-mode optical fibers that were connected to a spectrometer (Ocean Optics, Orlando, United States, LEDPRO-50) by a mechanical optical fiber switch. It should be noted that the switching time of the optical fiber switch is 15 ms, which did not affect the measurement efficiency of the experiments. A single-channel spectrometer can be employed to obtain spectra of two axes, due to the employment of the optical switch, which reduces the overall cost. Two compact linear stages (Physik Instrumente (PI) GmbH & Co. KG., Karlsruhe, Germany, M-112-1DG) with a scanning range of 25 mm and a positioning resolution of 50 nm were employed to move the single-mode optical fibers along the optical axes. The moving distances of the two linear stages were calibrated by the capacitive displacement sensor (MicroSense, LLC, Massachusetts, United States, Microsense II-5810) with a measurement range and resolution of 250 µm and 25 nm, respectively. Figure 10a shows the output spectrum (with normalized intensity) of the employed chromatic LED light source, and Figure 10b depicts two detected spectral signals obtained via the two fiber detectors at the two focal planes of the two dispersion lenses L1 and L2 with no off-axis distance by adjusting the end face of the two single-mode fibers fixed on the two compact linear stages. It should be noted that the established chromatic dispersion probe for the dual-axis absolute and relative displacement measurement can be extended to multiple simultaneous position sensors via multiple beam splitters.

### 3.2. FWHM Optimization

From the point of view of the equipment, the *Z*-directional measurement resolution of the proposed chromatic dispersion probe is mainly determined by the temporal and spatial stability of the employed light source spectrum, the signal-to-noise ratio during the whole measurement process, and the spectral resolution of the utilized optical spectrum analyzer [35,44]. From the perspective of the axial response, as one of the most important characteristics of the *Z*-directional measurement resolution of the proposed chromatic dispersion probe, the full width at half maximum (FWHM) of the axial response is the most commonly used indicator for evaluating the *Z*-directional measurement resolution [35,44].

**Figure 11:**
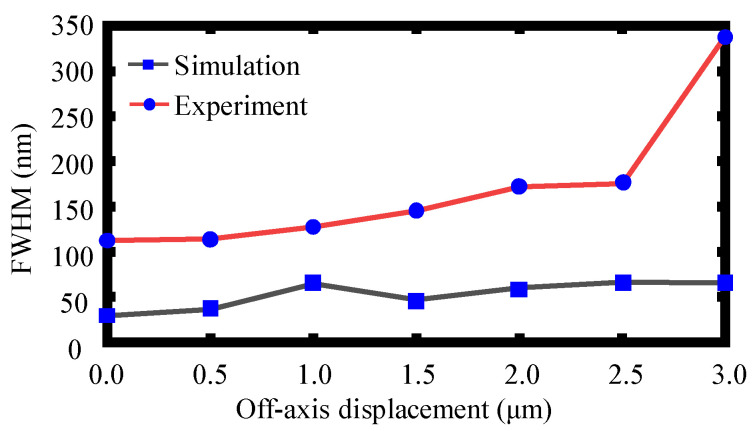
Simulation and experimental results of the FWHM values at different off-axis distances *D*.

To realize a better *Z*-directional measurement resolution, the minimum FWHM should be obtained among different off-axis distances *D* between the core center of the single-mode optical fiber and the center of the focal point of the dispersive lens. To obtain the minimum FWHM, the off-axis distance *D* was changed along the *X* direction and the corresponding FWHMs of the obtained spectral signals were approached. Figure 11 shows the simulation and experimental results of the FWHM varying with the off-axis distance *D.* As can be observed, the minimum FWHM can be obtained when *D* equals 0. Therefore, the off-axis distance *D* takes the value of 0 in the following simulations and experiments. Meanwhile, the defocus distance was used for displacement measurement of the proposed chromatic dispersion probe. It should be pointed out that, as can be seen in Figure 11, despite the FWHW values being different at the same off-axis displacement *D* in the simulation and the experiment, both the simulated and the experimental results achieve a minimal FWHM value when the off-axis displacement *D* was set to about 0.

### 3.3. Simulation and Discussions

#### 3.3.1. Simulation of the Absolute Displacement Measurement Range of Each of the Dual-Axes

**Figure 12:**
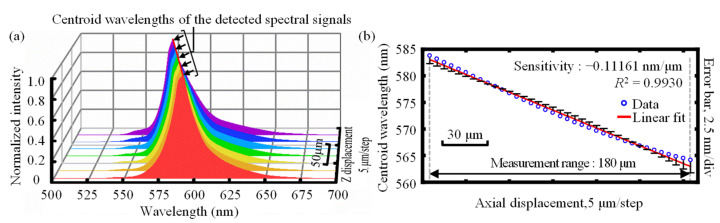
Simulation results of (**a**) the normalized output spectrum at each *Z*-axis displacement and (**b**) the absolute displacement measurement range of each of the dual-axes.

According to Equation (12), a simulation of the absolute displacement measurement range of each of the dual-axes of the established chromatic dispersion probe system was carried out. The light source used in the simulation is the employed light source in the experiment, whose spectrum is shown in Figure 10a. Figure 12 shows the simulation results in which the absolute displacement measurement range was approximately 180 μm, with the coefficient of determination *R*^2^ being 0.9930 and the measurement sensitivity being −0.11161 nm/μm, as is shown in Figure 12.

#### 3.3.2. Simulation of the Relative Displacement Measurement Range of the Dual-Axis

**Figure 13:**
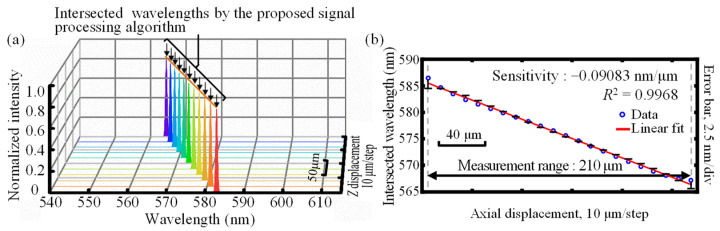
Simulation results of (**a**) the normalized output *I*_dual-axis_ by the proposed signal processing algorithm at each relative displacement of the dual-axis and (**b**) the relative displacement measurement range of the dual-axis.

According to Equation (21), Figure 13 depicts the simulation results of the relative displacement measurement range of the dual-axis of the established chromatic dispersion probe, in which the measurement range and sensitivity were about 210 μm and −0.09083 nm/μm, respectively, with the coefficient of determination *R*^2^ being 0.9968. It should be noted that to more accurately explain the experimental results, the actual light source spectrum detected in the experiment is employed in our simulation. However, in the experiment, due to the influence of the light source stability, temperature, and humidity, there exists spectral instability. Therefore, the center-of-mass method is employed in both the simulation and the experiments shown in Figure 12 and Figure 13. It should also be pointed out that in Figure 12 and Figure 13, with an employed pinhole with a radius of 25 μm, the measurement sensitivity can exhibit a small variation with different types of superimposed paraxial point light sources in theory. In our simulation, a radius range of 25 microns corresponded to the used 25 microns radius pinhole. A circle of point light sources is superimposed every 1 micron. Moreover, the simulation results are only a rough estimation of the absolute displacement measurement range of each of the dual-axes of the proposed chromatic dispersion probe system based on the linear fitting method that finds the centroid wavelength of the output spectra associated with different input axial displacement.

## 4. Experiments and Discussion

### 4.1. The Absolute Displacement Measurement of Each of the Dual-Axes

#### 4.1.1. Measurement Range and Sensitivity of Each of the Dual-Axes

**Figure 14:**
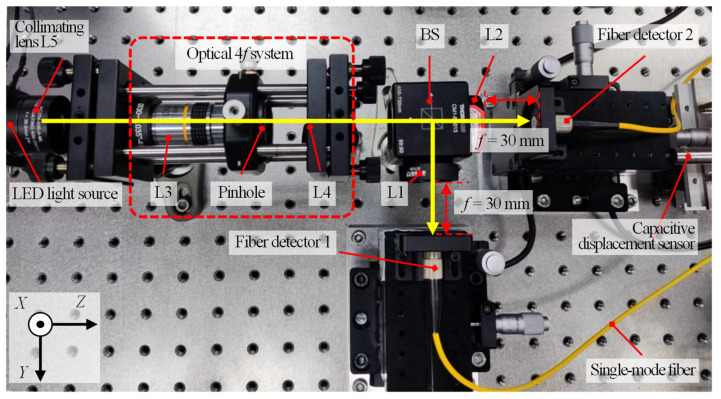
The practical optical configuration of the fiber-based chromatic dispersion probe for the simultaneous measurement of the dual-axis absolute and relative displacement.

To verify and confirm the feasibility and stability of the proposed chromatic dispersion probe, experiments were first carried out to investigate the actual absolute displacement measurement range and sensitivity of each dual-axis. Figure 14 illustrates the practical optical configuration of the fiber-based chromatic dispersion probe for dual-axis absolute and relative displacement measurement. The GCL-0101 series K9 flat-coated lenses (Daheng New Epoch Technology, Inc., Beijing, China) were used as the dispersive lenses in this system. The specific parameters of the dispersive lens and the Sellmeier coefficients of K9 are shown in Table 1 [42,45]. In these experiments, first of all, each end face of the two single-mode fibers of the two fiber detectors fixed on the two compact linear stages was made to scan axially with a moving interval of 5 μm until a linear relationship between the centroid wavelength of the obtained spectrum and the moving step was observed. Then, two fiber detectors were scanned with a moving interval of 5 μm simultaneously, and the corresponding spectra were obtained in which the axial displacement of the two compact linear stages was measured and calibrated simultaneously by the capacitance displacement sensors with a measurement range and resolution of 250 μm and 25 nm, respectively.

**Figure 15:**
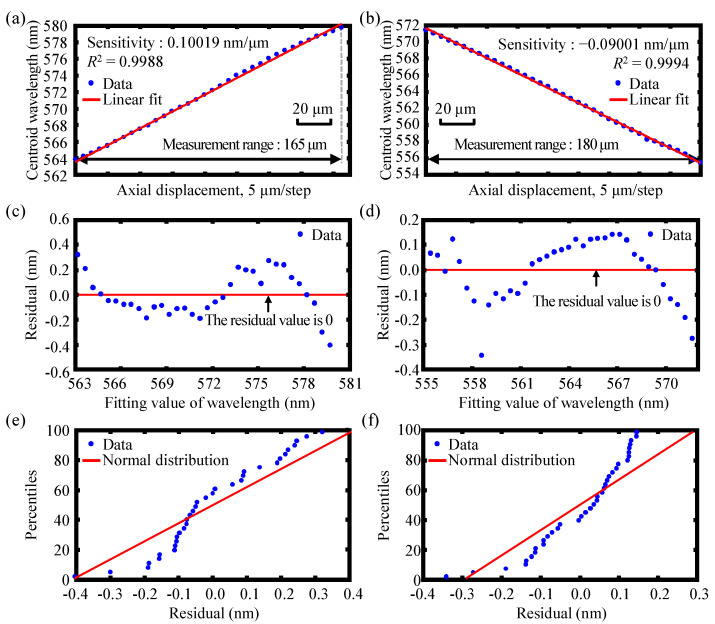
Experimental results of the measurement range for each of the dual-axes of the established fiber-based chromatic dispersion probe with a moving step of 5 μm in (**a**) axis 1 and (**b**) axis 2, the corresponding residual of the measurement range for (**c**) axis 1 and (**d**) axis 2, and the normal probability plot of residuals for (**e**) axis 1 and (**f**) axis 2.

Figure 15a,b show the experimental results of the measurement range and sensitivity for each of the dual-axes with a moving step of 5 μm, and the corresponding residual of the measurement range for the dual-axis are also presented in Figure 15c,d, respectively. Meanwhile, Figure 15e,f represent the normal probability plot of residuals for both axes, respectively. The measurement sensitivity of the established instrumental configuration was determined by the slope *k* and the coefficient of determination *R*^2^ of the fitted straight line. The larger the slope, the higher the sensitivity, and the closer the *R*^2^ to 1, the smaller the calibration error. For axis 1, as can be observed from Figure 15a,c, the fitted straight line yielded a measurement range and sensitivity of 165 μm and 0.10019 nm/μm, respectively, with the coefficient of determination *R*^2^ = 0.9988. For axis 2, Figure 15b,d present the fitted straight line, which yielded a measurement range and sensitivity of 180 μm and 0.09001 nm/μm, respectively, with the coefficient of determination *R*^2^ = 0.9994. It should be pointed out that the slopes are not the same because, after being divided into two sub-beams by the beam splitter, the angle of the two sub-beams cannot be completely vertical. Additionally, the intensity and the angle entering into the end face of the optical fiber in two axes are also slightly different, resulting in a little difference in the spectra of the two axes, and the slopes are not completely the same. It should also be pointed out that the end face of the optical fiber in fiber detector 1 moves in the direction away from the dispersive lens L1 in axis 1, and the end face of the optical fiber in fiber detector 2 moves in the direction close to the dispersive lens L2 in axis 2. Different moving directions of the fiber probes for axes 1 and 2 are chosen purposely to illustrate that two moving directions of the fiber probe measurement of the absolute displacement for both axis 1 and 2 can be realized. Furthermore, the relative displacement of the fiber probes on axis 1 and axis 2 can also be measured regardless of the moving directions of the fiber probes near or far away from the dispersive lenses L1 and L2. As can be seen in Figure 15, the experimental results of the measurement range and sensitivity of each of the dual-axes were in acceptable agreement with those of the simulation results in Section 3.3. It should be noted that Figure 15 was only a rough estimation of the simulated measurement range value of the proposed chromatic dispersion probe system based on the linear fitting method by finding the centroid wavelength of the output spectra associated with different input axial displacement. Moreover, the normal probability plots of the residuals of the measurement range shown in Figure 15e,f demonstrate that the distribution of residuals of the measuring points is normally distributed and is distributed in the 98% confidence interval. It should also be pointed out that the coefficient of determination *R*^2^ was employed in the simulation and experiment only as a qualitative evaluation of the goodness of fit of the linear regression and not as an indicator of the quantitative evaluation of the measurement linearity of the proposed chromatic dispersion probe. Furthermore, *R*^2^ was not the determination index of the measurement range of the proposed chromatic dispersion probe, and, as the next step of our research, work will be carried out to accurately evaluate the measurement range and measurement linearity with the accurate identification of the measurement accuracy and uncertainties.

#### 4.1.2. Measurement Resolution of Each of the Dual-Axes

**Figure 16:**
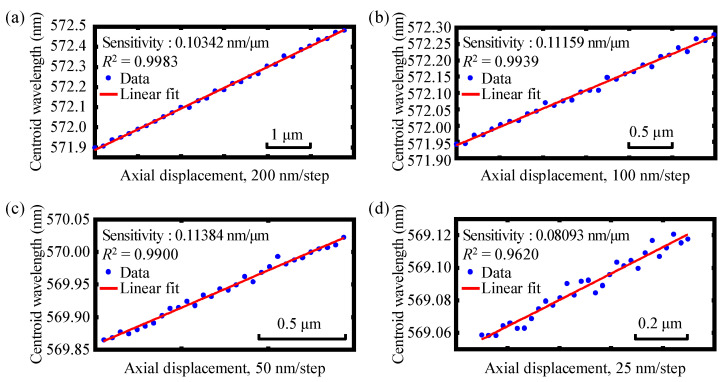
Experimental results of the absolute displacement resolution of axis 1 with different moving steps of (**a**) 200 nm, (**b**) 100 nm, (**c**) 50 nm, and (**d**) 25 nm, respectively.

**Figure 17:**
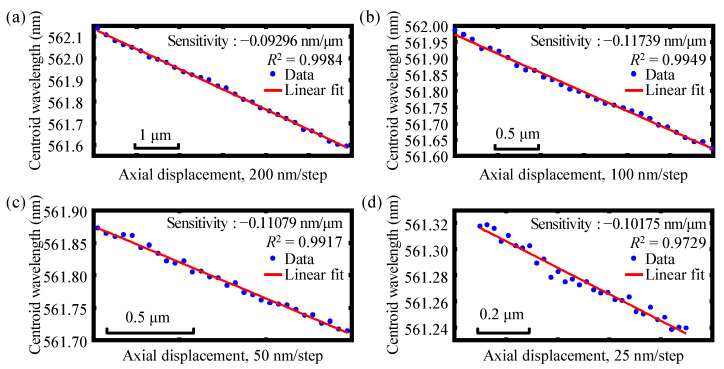
Experimental results of the absolute displacement resolution of axis 2 with different moving steps of (**a**) 200 nm, (**b**) 100 nm, (**c**) 50 nm, and (**d**) 25 nm, respectively.

Experiments were then performed to investigate the actual absolute displacement measurement resolution of each of the dual-axes. Similarly, in these experiments, each of the end faces of the two single-mode fibers of the fiber detectors mounted on the two compact linear stages were made to scan axially with a much smaller moving interval of 200 nm, 100 nm, 50 nm, and 25 nm, respectively. Figure 16a–d show the experimental results of the absolute displacement resolution of axis 1 with different moving steps of 200 nm, 100 nm, 50 nm, and 25 nm, respectively. The measurement resolution of axis 2 is illustrated in Figure 17. Through our experiments, it was found that when the step size was gradually reduced to 50 nm, good fitting linearity could easily be distinguished, and when the step size was reduced to 25 nm, which was achieved via piezo actuators (Physik Instrumente (PI) GmbH & Co. KG., P843-20) with a scanning range of 30 μm and a positioning resolution of 0.6 nm, the linear fitting results could not easily be distinguished and determined for either axes, 1 or 2. Hence, it can be concluded that greater than 50 nm of absolute measurement resolution can be approached using the established chromatic dispersion probe. As the next step in our research, further optimization and improvement of the measurement resolution of our instrument will be carried out.

### 4.2. The Relative Displacement Measurement of the Dual-Axis

#### 4.2.1. Measurement Range and Sensitivity of the Relative Displacement Measurement of the Dual-Axis

**Figure 18:**
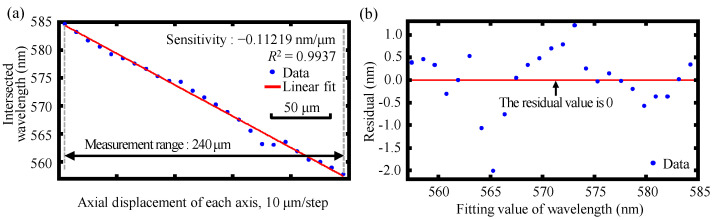
Experimental results of (**a**) the measurement range and sensitivity together with (**b**) the corresponding residual of the dual-axis relative displacement measurement of the established fiber-based chromatic dispersion probe with a moving step of 5 μm.

Experiments were further performed to verify and confirm the feasibility and basic performance of the relative displacement measurement of the dual-axis of the proposed chromatic dispersion probe. In the experiments, the two fiber detectors were scanned in opposite directions with 5 μm steps by biaxial synchronization, and the corresponding spectral signals were recorded simultaneously. The relative displacement of the dual-axis could be derived and extracted by the proposed signal processing algorithm aimed at finding the position of the spectral intersection of the two detected spectral signal curves. Figure 18 presents the experimental results of the measurement range and sensitivity together with the corresponding residual of the relative displacement measurement of the dual-axis of the established chromatic dispersion probe, which yielded a relative displacement measurement range of 240 μm and sensitivity of −0.11219 nm/μm, respectively, with a coefficient of determination *R*^2^ of 0.9937. As can be observed in Figure 18, the experimental results of the measurement range of the relative displacement measurement of the dual-axis were also in acceptable agreement with the simulation results in Section 3.3. It should be noted that due to the spectral instability and thermal drift during the experiment, the measuring sensitivity was slightly smaller than that of the simulation. It should also be pointed out that since the light beam was divided into two sub-beams at 90 degrees by the BS, the measurement of the absolute and relative displacement of the dual-axis at 90 degrees was carried out in this paper. In our future work, the dual differential confocal method [46] will be taken into consideration to optimize the proposed chromatic dispersion probe and to achieve better performance of the measurement range.

#### 4.2.2. Measurement Resolution of the Relative Displacement Measurement of the Dual-Axis

**Figure 19:**
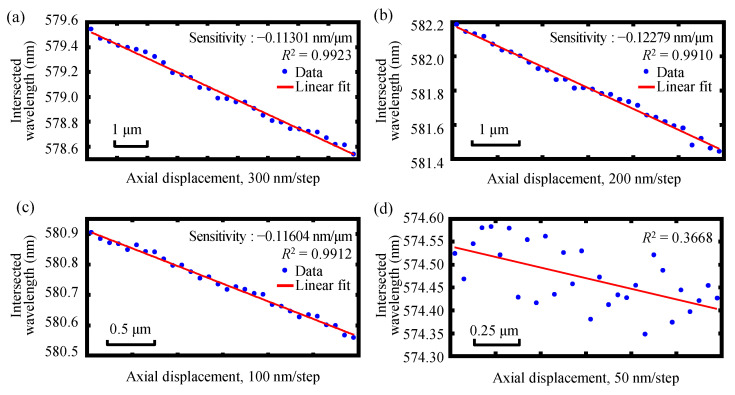
Experimental results of the relative displacement resolution of the dual-axis of the developed fiber-based chromatic dispersion probe with different moving steps of (**a**) 300 nm, (**b**) 200 nm, (**c**) 100 nm, and (**d**) 50 nm, respectively.

Experiments were then carried out to further investigate the biaxial relative displacement resolution, in which each of the end faces of the two single-mode fibers of the two fiber detectors were made to scan synchronously in opposite directions with a relative moving interval of 300 nm, 200 nm, 100 nm, and 50 nm, respectively. Figure 19 shows the measurement resolution of the relative displacement measurement of the dual-axis of the established chromatic dispersion probe. As can be seen in Figure 19, the applied moving step cloud still be easily distinguished, and good fitting linearity could be observed when the step size was reduced to 100 nm. In contrast, when the moving step approached 50 nm, it was not easy to maintain good linearity, and the deviation increased significantly, which indicates that the measurement resolution of the relative displacement measurement of the dual-axis was better than 100 nm. It should be noted that the dual-axis of our established chromatic dispersion probe could not only move in the opposite direction relative to the focal planes of the two dispersive lenses L1 and L2, just as in our relative displacement measurement experiments, but the dual-axis could also move in the same direction. Due to the spectral instability, the peak wavelength was replaced by the centroid wavelength in the data processing of the simulation and experiment results for absolute displacement measurement. Meanwhile, a signal processing algorithm was proposed for the relative displacement measurement that tracked the intersection of two detected spectra from two fiber detectors rather than the peak wavelength; that is to say, for the relative displacement measurement, the peak wavelength was not used for any measurement process. Thus, there was no clear correspondence between the measurement resolution for absolute displacement measurement and the measurement resolution for relative displacement measurement. In our experiments, the absolute displacement resolution of a single axis could reach 50 nm. However, when two axes moved at the same time, there were measurement errors in both axes. Therefore, the relative displacement resolution would be less than the absolute displacement resolution theoretically, and it was twice as much as the absolute displacement resolution of 50 nm. Future work will also be carried out to further optimize and improve the relative displacement measurement resolution of the dual-axis of our instrument.

## 5. Conclusions

A novel fiber-based chromatic dispersion probe for the simultaneous measurement of dual-axis absolute and relative displacement with nanometric resolutions has been proposed and developed. The proposed chromatic dispersion probe is based on optical dispersion in such a way that the employed light beam is divided into two sub-beams that are then made to pass through two optical paths with different optical settings where two identical single-mode fiber detectors are located at different defocused positions, respectively, aimed at obtaining two spectral signals that are used to measure the absolute displacement of the dual-axes, respectively. A signal processing algorithm is proposed to obtain a spectral intersected wavelength that indicates the relative displacement of the dual-axis. With the proposed chromatic dispersion probe, the absolute and relative displacement measurement of the dual-axis can be realized simultaneously, and the sum of Sin fitting and biaxial height matching methods are used to improve the linearity and resolution of the relative displacement measurements of the dual-axis. Theoretical and experimental investigations have revealed that the developed chromatic dispersion probe realizes an absolute measurement range and a measurement resolution of approximately 180 μm and 50 nm, respectively, for each axis, and further approaches a relative displacement measurement range and a measurement resolution of about 240 μm and 100 nm, respectively, for the dual-axis.

It should be pointed out that this paper is an initial report on the first step of our research on the establishment of a fiber-based chromatic dispersion probe for the simultaneous measurement of the dual-axis absolute and relative displacement. As the next step of our research, the optimization of the proposed chromatic dispersion probe and the achievement of a longer measurement range, as well as a higher measurement resolution, will be taken into consideration. Furthermore, a more detailed investigation into the measurement accuracy/uncertainty of the proposed chromatic dispersion probe, as well as the identification technology of the optical frequencies/wavelengths of the used light source, will also be contained in the next step of our research.

## Figures and Tables

**Table 1:** Physical parameters of the employed dispersive lenses L1 and L2.

Items		Symbol	Value
Lens materials		-	K9
Surface radii of the lenses L1 and L2		*R*_1_	∞
		*R*_2_	15.5 mm
Central wavelength of the dispersive lenses L1 and L2		*λ*_central_	558 nm
Sellmeier coefficients			
*B*_1_	1.03961212	*C*_1_	0.00600069867
*B*_2_	0.231792344	*C*_2_	0.0200179144
*B*_3_	1.01046945	*C*_3_	103.560653

## Data Availability

Not applicable.
